# The Role of Kisspeptin in the Control of the Hypothalamic-Pituitary-Gonadal Axis and Reproduction

**DOI:** 10.3389/fendo.2022.925206

**Published:** 2022-06-28

**Authors:** Qinying Xie, Yafei Kang, Chenlu Zhang, Ye Xie, Chuxiong Wang, Jiang Liu, Caiqian Yu, Hu Zhao, Donghui Huang

**Affiliations:** ^1^ Institute of Reproductive Health, Tongji Medical College, Huazhong University of Science and Technology, Wuhan, China; ^2^ Department of Human Anatomy, Tongji Medical College, Huazhong University of Science and Technology, Wuhan, China

**Keywords:** kisspeptin, KISS1R, HPG axis, KNDy neurons, reproduction

## Abstract

The discovery of kisspeptin as a critical central regulatory factor of GnRH release has given people a novel understanding of the neuroendocrine regulation in human reproduction. Kisspeptin activates the signaling pathway by binding to its receptor kisspeptin receptor (KISS1R) to promote GnRH secretion, thereby regulating the hypothalamic-pituitary-gonadal axis (HPG) axis. Recent studies have shown that kisspeptin neurons located in arcuate nucleus (ARC) co-express neurokinin B (NKB) and dynorphin (Dyn). Such neurons are called KNDy neurons. KNDy neurons participate in the positive and negative feedback of estrogen to GnRH secretion. In addition, kisspeptin is a key factor in the initiation of puberty, and also regulates the processes of female follicle development, oocyte maturation, and ovulation through the HPG axis. In male reproduction, kisspeptin also plays an important role, getting involved in the regulation of Leydig cells, spermatogenesis, sperm functions and reproductive behaviors. Mutations in the *KISS1* gene or disorders of the kisspeptin/KISS1R system may lead to clinical symptoms such as idiopathic hypogonadotropic hypogonadism (iHH), central precocious puberty (CPP) and female infertility. Understanding the influence of kisspeptin on the reproductive axis and related mechanisms will help the future application of kisspeptin in disease diagnosis and treatment. In this review, we critically appraise the role of kisspeptin in the HPG axis, including its signaling pathways, negative and positive feedback mechanisms, and its control on female and male reproduction.

## 1 Introduction

Reproduction is essential to the survival of all species. The hypothalamic-pituitary-gonadal (HPG) axis which consists of anterior hypothalamus, pituitary, and the gonads plays an indispensable role in regulating reproduction in human and other animals (1). Female follicular development, egg maturation, ovulation and spermatogenesis in male are highly driven by HPG axis (2). As the promoter of HPG axis, the gonadotropin-releasing hormone (GnRH) neurons connect to their neuronal network and send projections to the median eminence (ME) after they travel to medial preoptic area (POA) (3, 4). They are final output cells of the neuronal network that controls secretion of gonadotropins by the pituitary gland in all mammals (5). However, it is well known that GnRH neurons lack estrogen receptor α and progesterone receptors, which indicates that the positive and negative feedback effect of estrogen and progesterone on the gonadal axis is not directly on GnRH neurons, but mediated by other neurons (6).

Kisspeptin is a neuropeptide which was first discovered by Lee et al. in 1996 (7). It was later found that it acts through the G-protein-coupled receptor, GPR54 (8). Although kisspeptins were first considered to be related to cancer metastasis, they were later found be closely related to reproduction, in which they modulate GnRH secretion, thus exerting a significant role in the regulation of the HPG axis (9, 10). Later, as the reproductive role for neurokinin B was discovered, kisspeptin was confirmed to be associated with positive and negative feedback of estrogen. In 2006, scientists found that proNKB was expressed in most prodynorphin-ir neurons in the rats arcuate nucleus (ARC), and almost all proNKB neurons are immunoreactive to prodynorphin, indicating that there is a close relationship between Dyn and NKB (11). The location of kisspeptin, neurokinin B(NKB) and dynorphin (Dyn) in the hypothalamus are overlapped and frequently colocalized in a very conserved region across species (11–13). By regulating the secretion of NKB and Dyn, ARC kisspeptin neurons modulate kisspeptin expression, thereby mediate the negative feedback of sex steroids (14). Besides, kisspeptin neurons which located in rostral periventricular region of the third ventricle (RP3V) (in rodents) and preoptic area (POA) (in human) regulate estradiol positive feedback by expressing ERα and cotransmitters such as tyrosine hydroxylase, GABA and glutamate, with the latter two have the effect of exciting GnRH neurons (15). These findings made the understanding of the HPG axis more complete and stimulated further interest in the field of the effect of kisspeptin in reproduction.

Since the relationship between kisspeptin and reproduction was reported, a large number of studies has been conducted about the regulatory role of kisspeptin in the HPG axis and its detailed mechanisms (10, 16–18).Besides, more and more studies have shown that kisspeptin, as an upstream regulator of HPG axis, plays an indispensable regulatory role in the beginning of puberty and participates in various processes of female reproduction whether in animals or human (9, 19). The function of kisspeptin in male reproduction have also been studied in a large number of amphibians, fish, rodents and primate, while it is still less clear (20–23). The increasing understanding of kisspeptin promotes the application of kisspeptin in clinical diagnosis and disease treatment. In this review, we summarize current understanding of the regulatory role of kisspeptin on HPG axis and discuss the effects of central and periphery kisspeptin on male and female reproduction.

## 2 Kisspeptin and Kisspeptin Receptor

### 2.1 Kisspeptin

In 1996, *KISS1* gene were first found by Lee et al. when they were when studying different metastatic abilities of human melanoma cells (7). Interestingly, because it was discovered at the place where Hershey’s chocolate was produced, its name came from a kind of Hershey’s chocolate named kisses. In humans, *KISS1* is located on the long (q) arm of chromosome 1 at q32 with four exons (7, 24). The KISS1 transcription start site (TSS) is located between -153 bp to -156 bp upstream of the ATG in human (25). The promoter of KISS1 contains numerous GC-rich elements, which are the binding sites of Sp1 family proteins. Sp1 and Sp3 are universal transcription factors which function by forming dimers. Sp1 activates the transcription while Sp3 represses it, thus the ratio of Sp1 and Sp3 is crucial for the differential expression of *KISS1* in different tissues (26). The GC-rich motifs are also the targets of estrogen induced kisspeptin expression. Other regulators include Transcription Factor 1 (TTF1), ectoderm development (EED), Chromebox protein homolog 7 (CBX7) and so on (25). Among the four exons, the first two exons do not encode any peptides and the remaining two can be partially translated, encoding a precursor peptide containing 145 amino acids (24). The precursor, which has a 19 amino acid signal peptide, can further yield four short peptides, namely kisspeptin-54 (kp-54), kisspeptin-13 (kp-13), kisspeptin-10 (kp-10), distinguish by the number of amino acids, respectively (27, 28). These peptides are collectively referred to as kisspeptins, in which kp-54 is the main product. Kisspeptins belong to the RF amide peptide family, with special Arg – Phe – NH2 motifs in their C-terminal region, which contribute to their binding to kisspeptin receptor (KISS1R) (27).

The location of kisspeptin neurons are different between rodents and human. In rodents, kisspeptin neurons were discovered in ARC and the anteroventral periventricular nucleus (AVPV) which extends into the periventricular nucleus (PEN) (10, 29, 30). The latter two nucleus are also known collectively as RP3V (31). In humans, kisspeptin neurons were distributed predominantly in the infundibular nucleus and sparsely in POA (32). Interestingly, kisspeptin neurons within the rodent RP3V have obvious difference between male and female. The number of kisspeptin neurons in female rodents is prominently bigger than that in the male (33). In humans the situation is similar as studies have observed that there are much more kisspeptin fibers in the infundibular nucleus and rostral preoptic area in women than in men (34).

Apart from these places, kisspeptin can also be found in limbic and paralimbic brain regions in extra-hypothalamic areas and placenta, pancreas, ovary and liver in peripheral areas (33, 35–37). In rat and human ovaries, *Kiss1* mRNA is primarily detected in granulosa cells, indicating that ovarian kisspeptins are possibly synthesized by granulosa cells (38, 39).

### 2.2 Kisspeptin Receptor

Kiss1R, first known as G-protein coupled receptor 54 (GPR54), AXOR12 or hOT7T175, was discovered in the rat brain in 1999 as an orphan receptor (17, 40). Being a G-protein-coupled receptor of seven transmembrane domains, KISS1R is a kind of Gq/11 protein-associated receptor. It was not until 2001 when GPR54 was identified as a putative receptor for *KISS1*-derived peptides (35, 36). In human, *KISS1R* is located on chromosome 19p13.3 and encodes a protein with 398 amino acids, which can be detected in the cerebral cortex, thalamus, pons-medulla and cerebellum in central areas (40, 41). In rodents, kisspeptin receptor is expressed in POA and ARC in hypothalamus and hippocampus outside of the hypothalamus (27, 35, 36, 40, 42). In fact, most GnRH neurons express *Kiss1R* mRNA in both young and adult mice (43). In the periphery, *KISS1R* mRNA exists in plenty of tissues in human, including pancreas, stomach, small intestine, thymus, spleen, lung, gonads and so on (35, 36). In human ovary, KISS1R is expressed in corpus luteum, parietal granulosa cells, granulosa cells, theca cells, oocytes, stromal cells and epithelial cells (44). Unlike *Kiss1* mRNA is mainly expressed in granulosa cells, KISS1R expression is obviously higher in oocytes than that in granulosa cells (45). Also, KISS1R is expressed in human endometrial stromal cells and placental trophoblasts (46, 47).

### 2.3 Kisspeptin Signaling Pathway

**Figure 1:**
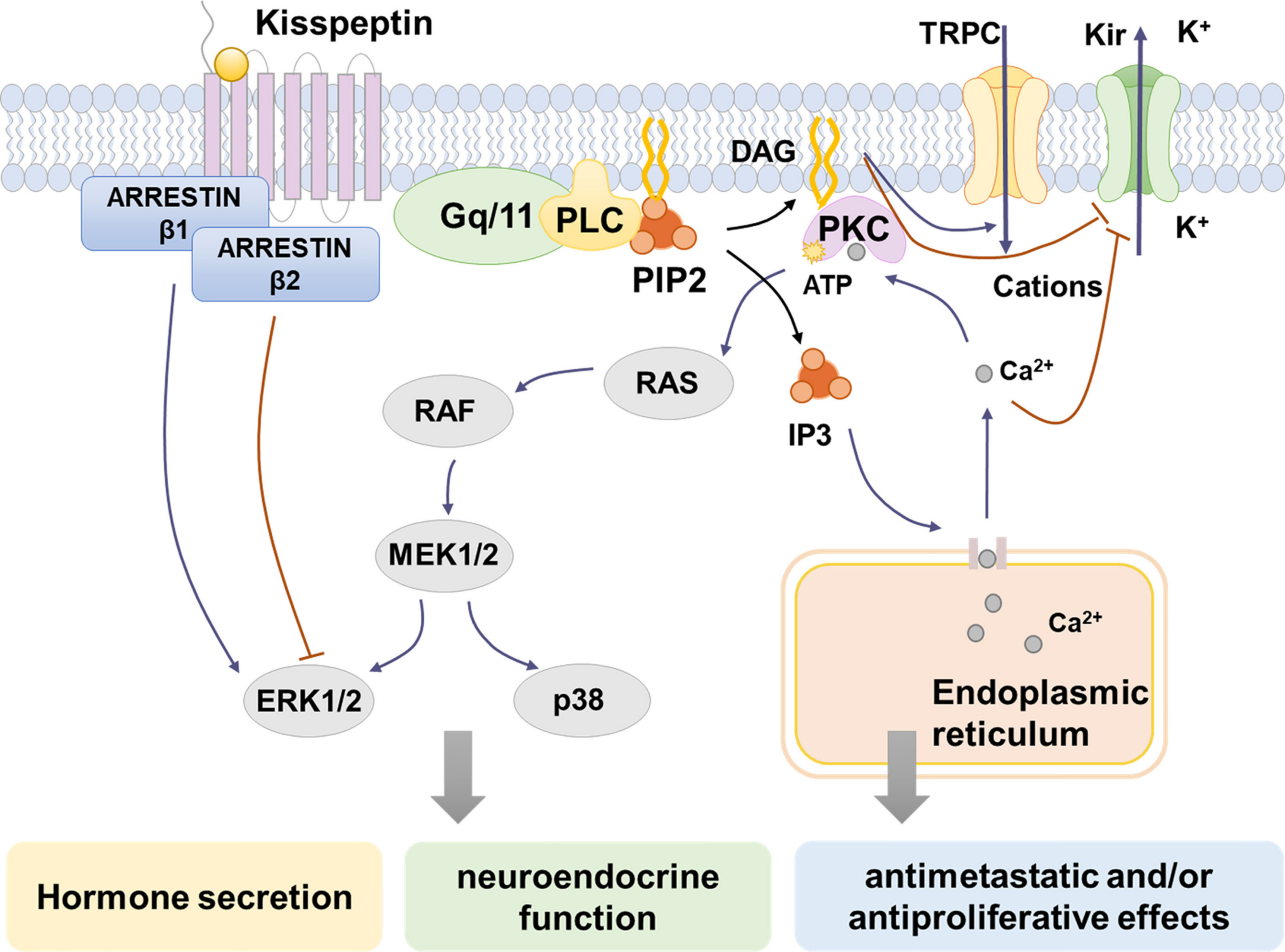
Kisspeptin/KISS1R signaling pathways. Kisspeptin binds to KISS1R, inducing the intracellular portion of KISS1R phosphorylates Gq/11. Then PLC is activated and it hydrolyzes PIP2 to IP3 and DAG. IP3 induces intracellular Ca^2+^ release from the endoplasmic reticulum, while DAG activates PKC, causing the phosphorylation of MAPK, such as ERK1/2 and p38. Moreover, the activation of KISS1R recruits arrestin-1 and -2, which decreases and increases ERK1/2 phosphorylation, respectively. The increase of intracellular Ca^2+^ changes ion channel permeability by block the inwardly rectifying potassium channel (Kir). The depolarization of GnRH neurons is caused by activation of a nonselective cation channel (TRPC) and suppression of Kir by DAG and increased Ca^2+^. Through the signaling pathways above, kisspeptins activate different MAPKs and cause the release of Ca^2+^, which contribute to hormone-releasing regulation, neuroendocrine function, antimetastatic and/or antiproliferative effects.

After kisspeptin binds to KISS1R, the activated G protein-coupled receptor will be dissociated into Gα_q/11_-GTP and Gβγ (48). Gα_q/11_ then activate phospholipase C (PLC)-β, a key enzyme in the cytoplasm. Under the action of PLC-β, phosphatidylinositol 4, 5-bisphosphate (PIP2) is hydrolyzed to produce inositol- (1, 4, 5)-triphosphate (IP3) and diacylglycerol (DAG). IP3 then combines with the IP3-sensitive Ca^2+^ channel, inducing intracellular Ca^2+^ mobilization and release, which can promote various different functions depending on the cell context (27, 44, 49). An increase in Ca^2+^ could convey the ability of hormone secretion of kisspeptin and regulate its ability to inhibit cell proliferation (50). DAG activates protein kinase C, which subsequently causes phosphorylation signaling cascade and finally leads to the activation of mitogen-activated protein kinases (MAPKs), such as ERK1/2 and p38 (27) **(** **Figure 1** **)**. However, the ERK1/2 and p38 activation caused by kisspeptin treatment varies between different cell types (50). In Chinese hamster ovary K1 cells treated with kisspeptin-10, ERK1/2 showed strong continuous phosphorylation, while p38 showed weak phosphorylation (27). Likewise, kisspeptin can stimulate ERK1/2 activation while P38 MAPK is not affected in cells from rat corpus luteum (51) and anaplastic thyroid cancer cells (52). In HT-1080 cell line, excessive expression with kisspeptin diminished synthesis of matrix metalloproteinase-9 (MMP-9) enzyme activity by inhibiting the translocation of nuclear factor κB (NF-κB), thereby decreasing promoter binding of NF-kB to the MMP-9 promoter (53). DAG also activates a nonselective cation channel (TRPC) and inhibits an inwardly rectifying potassium channel (Kir) at the same time, thus causing continuous depolarization of GnRH neurons and stimulates GnRH release (54). Additionally, activated KISS1R can recruit β-arrestin-1 and -2 to the membrane, which assist with KISS1R signaling (55, 56). β-arrestin-1 and β-arrestin-2 have contrast effects, while β-arrestin-1 promotes the phosphorylation of ERK1/2 while the other inhibits the process. Other studies have also confirmed that β-arrestin-1 and -2 play a role in modulating the ERK1/2 phosphorylation in different kinds of cells (57–59).

Thus, as mentioned above, kisspeptins stimulate KISS1R through a variety of signaling pathways. In different cell types, activation of KISS1R induces phosphorylation of different kinds of MAPK, which finally take part in hormone-releasing regulation, neuroendocrine function, antimetastatic and/or antiproliferative effects of kisspeptins (50).

## 3 The Function of Kisspeptin in HPG Axis

### 3.1 Kisspeptin Stimulates Gonadotrophin Release

In both animals and humans, kisspeptin is a powerful stimulator of the HPG axis (17, 60). In 2003, Seminara et al. first conducted studies to explore the relationship between kisspeptin and reproductive axis in mice (9). They found that injecting GnRH into kiss1r deficient mice improved their hypogonadotropic symptoms, suggesting that the effect of kisspeptin was mediated by stimulating GnRH release. In the following two years, several studies have reported the underlying promoting effect of kp-10 and kp-54 on LH secretion in rodents respectively (10, 61–63), providing preliminary evidence for the effects of kisspeptin in the process of GnRH release. More details have been found gradually since 2005. Neuroanatomical studies have confirmed that a large number of GnRH neurons were distributed in POA, and more than 90 percent of GnRH neurons express Kiss1R in mice (43). Projections from kisspeptin neurons to GnRH neurons are different between species (15). In mice, AVPV kisspeptin neurons fibers project directly to GnRH cell bodies, while ARC kisspeptin neurons are primarily apposed to GnRH processes which extend to the median eminence (15). In human, kisspeptin neurons in the hypothalamus project fibers into POA, stimulating GnRH secretion which later acts on the pituitary and leads to LH and FSH release (64, 65). Experiments have confirmed that central and peripheral addition of exogenous kisspeptin stimulates this reproductive cascade in both human and animals (63, 66). Additionally, the use of a GnRH antagonist blocks the promoting effect of kisspeptin (10), demonstrating that kisspeptin sits at the top of the HPG axis. In recent years, the application of modern methods confirmed the role of kisspeptin more directly. Using an optogenetic approach, scientists have found that the synchronous activation of ARC kisspeptin neurons *in vivo* resulted in pulsatile LH release in rodents in a sex steroid-dependent manner (67, 68). By transfecting *Kiss1* to *Kiss1* knockout rats, Nagae et al. demonstrated the ARC KNDy neurons were the generator of GnRH pulse and the presence of more than 20% of ARC KNDy neurons was able to rescue reproductive function (69).

Furthermore, it is important to notice that the production and secretion of FSH and LH are affected by different GnRH pulse frequencies and amplitudes, which is associated with various signaling pathways. Low frequency of GnRH pulses is conducive to FSH secretion, while high frequency of GnRH pulses tends to cause the release of LH. In detail, high GnRH pulse frequency can activate the PKC/MAPK and Calcium/Calmodulin-dependent kinase II (CaMK II) pathways and stimulate early growth response-1 protein (Egr1) expression largely, thereby increasing Lhb transcription and producing more LH. In contrast, low frequency of GnRH pulses helps recruit the histone acetyltransferase CREB-binding protein (CBP) to the promoter, which stimulates Fshb transcription (70). At present, LH is as an alternative marker for the effect of kisspeptins on GnRH secretion. However, few studies have researched the role of kisspeptin in regulating FSH secretion, which may be attributed to the difficulty in accurate continued measurement of serum FSH concentration. Taken together, the studies above indicate that kisspeptin acts as a gatekeeper in the HPG axis, controlling the onset of HPG axis by stimulating GnRH secretion, and is related to LH pulsatile secretion (70).

### 3.2 Kisspeptin Mediates Negative and Positive Sex Steroids Feedback

**Figure 2:**
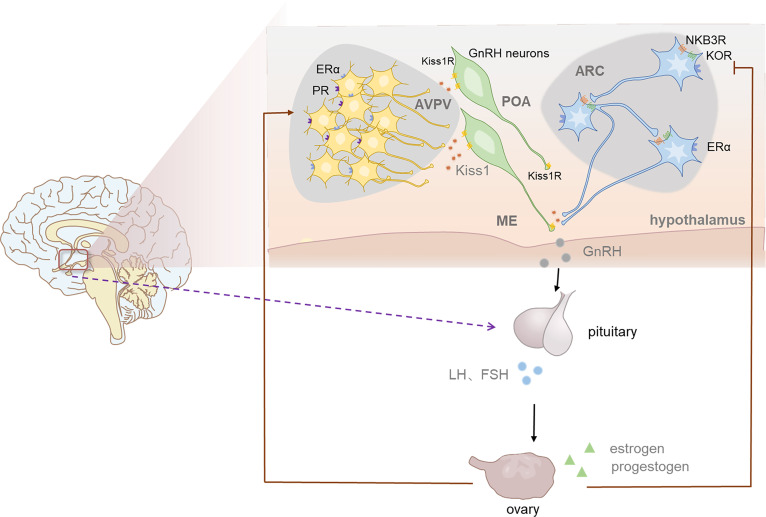
The role of kisspeptin in HPG axis and the positive and negative feedback of sex steoids in female. The population of kisspeptin neurons are located in anteroventral periventricular nucleus (AVPV) and the arcuate nucleus (ARC) in rodents. ARC kisspeptin neurons which coexpress NKB and Dyn are named KNDy neurons. Acting in an autocrine/paracrine manner, NKB stimulates kisspeptin secretion while Dyn inhibit it, thus regulate the release of GnRH indirectly. In this way KNDy neurons mediate negative feedback of estrogen and enables the generation and termination of GnRH pulse. AVPV NEURONS regulate the generation of GnRH sulge, thus induce LH surge, which is more significant in female ovulation. Progesterone also plays an indispensable role in the generation of GnRH surge.

Apart from the stimulation of kisspeptin on GnRH, some studies also explored the expression of *Kiss1* mRNA in ARC and RP3V respectively when exposed to sex steroids (71). The regulation in these regions were found to be different, as estradiol stimulates RP3V kisspeptin neurons while inhibits ARC kisspeptin neurons. In addition, scientists found that during LH surge, the level of *KISS1* mRNA was raised in RP3V but was reduced in ARC (72). These studies initially revealed that negative feedback is mediated by ARC kisspeptin neurons, while positive feedback is modulated by kisspeptin neurons in RP3V. The detailed mechanism has been deeply studied in the last two decades. According to the model built by scientists, NKB acts on KNDy neurons to cause pulsatility and Dyn acts as the ‘brake’ halting pulses and kisspeptin is the final output to GnRH neurons **(** **Figure 2** **)**. NKB acts in an autocrine/paracrine manner, inducing an inward current to increase the membrane potential of KNDy neurons, thus increasing the firing frequency of KNDy neurons and causing increased Ca^2+^ oscillations which finally promote the secretion of kisspeptin (73). The generation and termination of GnRH pulses are closely related to the change in the balance between stimulation (NKB) and inhibition (Dyn) tones, namely the ratio of NKB to Dyn (73). The model has been supported by mammal experiments and human studies. For example, ovariectomy increased *NKB* expression in ARC of monkeys, while estrogen treatment reduced it (74, 75). Post-menopausal women were found to have higher expression of *Kiss1* mRNA (32). The hypertrophied neurons in their infundibular nuclei expressed both ESR1 (encoding ERα) and NKB mRNA, showing a similar distribution to that of kisspeptin neurons (76, 77). Also, prodynorphin mRNA expression in the ARC decreased, showing the mediating role of dynorphin in sex steroid negative feedback. In general, humans KNDy neurons may mediate negative sex steroid feedback in the infundibular nucleus by inhibiting the secretion of kisspeptin and NKB and stimulating the secretion of Dyn, causing reduced activity of the GnRH neuronal system, thus affects the secretion of GnRH (18). In recent years the application of optogenetics, GCaMP fiber photometry and mathematical models have enabled scientists to observe direct activities of neurons, perform long-term recordings and understand their complex dynamic behaviors (78–80). High-frequency photo stimulation of ARC kisspeptin neurons induced release of NKB and Dyn (80). In a latest research, Moore et al. observed KNDy neurons activity at a single cell level through *in vivo* calcium imaging and found the activation of KNDy neurons was in a temporal order (81). According to their study, some subsets of KNDy cells act as “leaders” and others serve as “followers” during each synchronized episode. These results offer more direct evidence on the model.

Additionally, ARC kisspeptin neurons are important targets for progesterone feedback since progesterone receptor is expressed in almost all ARC kisspeptin neurons, while only a few is expressed in GnRH neurons (82, 83). As a neurosteriod, the level of progesterone dramatically increased after ovulation, which plays a role in inhibiting LH secretion. A study conducted in ewes has shown that the use of progesterone receptor antagonist in ARC kisspeptin neurons blocked the inhibiting effect of progesterone on LH pulse while the same effects did not occur when applying antagonist in POA (84). However, there are some differences in this model between different species. For example, the importance of NKB appears to be different between mice and human. NKB receptor-deficient mice show the same reproductive ability as wild type mice while human with NKB/NKB receptor mutations are reproductive incompetence (85–88). Moreover, although KNDy neurons express both estrogen and progesterone receptors, it seems that Dyn only mediates part of estrogen negative feedback in rodents, also (13, 89).

By contrast, AVPV kisspeptin neurons are more related to the positive feedback of estrogen. Estrogen feedback changes from negative to positive in the end of follicular phase to mediate the LH peak before ovulation. Emerging data suggest that AVPV kisspeptin neurons are more site and species specific and more sexually dimorphic (18). More than 70% AVPV kisspeptin neurons are ERα positive and express cFos, an immediate early gene whose expression represents increased neuronal activity during the LH surge (15, 33, 83, 90). A research found that in adult female mice more than one-third of AVPV kisspeptin neurons connect to GnRH cell bodies and nearly all of them express ERα (91). During the LH surge, AVPV kisspeptin neurons were observed higher level of cFos expression, which is an indication of increased activity of AVPV kisspeptin neurons (92). Experiments also confirmed that AVPV kisspeptin neurons are more active during estrogen positive feedback than negative feedback, as these neurons firing more action potentials and present stronger rapid action potential bursts on proestrus compared to other periods (93, 94). From these results collectively, preliminary conclusion could be drew that estrogen regulates AVPV kisspeptin neurons positively and increasing their activity, thus releasing GnRH stimulator kisspeptin and other cotransmitters, which induce the GnRH/LH surge. Besides, It is now established that progesterone receptors (PR), especially those expressed on kisspeptin neurons, are essential for positive feedback induced LH surge, since female mice lacking progesterone receptors exclusively in kisspeptin cells did not yield obvious LH surges when exposed to an estradiol-positive feedback paradigm (95–97). AVPV kisspeptin neurons coexpress PR, suggesting that they are targets for progesterone (96). A recent study found that selective reintroduction of PR into AVPV kisspeptin neurons, with other kisspeptin cells remaining PR absent, could rescue the LH surge in OVX (ovariectomized) + E_2_ females, indicating that PR in AVPV Kisspeptin neurons are enough for positive feedback induction of the LH surge (97).

Interestingly, despite ERα signaling in AVPV kisspeptin neurons is crucial for positive feedback, ERα signaling is not a necessary condition for negative feedback in ARC kisspeptin neurons, because mice with kisspeptin cells specifically lack ERα still retain negative feedback after estradiol therapy (98). This indicates that there is a redundant negative feedback path independent of kisspeptin signaling. In male, the number of AVPV kisspeptin neurons is very small (90). This sex-specific difference is determined by gonadal hormones during early neonatal development, in which neonatal testicular androgen leads to the decrease of AVPV kisspeptin expression (99). In male, ERα and androgen receptor (AR) are both expressed in most kisspeptin neurons of the two main kisspeptin neuronal populations (100). Compared with females, the detailed signaling mechanisms that mediate the testosterone feedback seem to have some differences between ARC and AVPV kisspeptin neurons. In AVPV, the effects of testosterone seem to be mediated by ERα or ERβ, since estradiol treatment is able to fully mimic the effect of testosterone (100, 101). In contrast to AVPV, ARC kisspeptin neurons do not show notable degree of sexual dimorphism (102). In conclusion, only the pulsatile LH release is evident in male mammals, and androgen negatively feedbacks to regulate the GnRH/LH pulse in order to maintain sustained spermatogenesis and steroidogenesis. The function of kisspeptin in the male AVPV is still unclear at present.

## 4 The Role of Kisspeptin in Reproduction

### 4.1 Kisspeptin Regulates Pubertal Onset

Puberty is described as the development of secondary sexual characteristics, the maturation of gonads, and the acquisition of reproductive competence (103). The process of puberty is complex and associated with thousands of genes (5). During the onset of puberty, the pulsatile release of GnRH from the hypothalamus plays a major role, which is the result of a couple of excitatory and inhibitory factors. For example, the excitatory factors include catecholamines and glutamate while the inhibitory factors include gamma-aminobutyric acid (GABA) and Opioid peptide (103). Among these excitatory factors, it is well known that the kisspeptin occupies an indispensable position. The *KISS1* mRNA expression increases significantly when people transit from juvenile to adult in AVPV although the expression of *KISS1R* mRNA does not have a detectable change in mice (43). The underlying mechanism involves reduction of EED and Cbx7, dissociation of Polycomb Group (PcG) and recruitment of trithorax Group (TrxG) elements at the Kiss1 promoter, which finally increases Kiss1 transcription (104, 105). In females, the late development of the POA kisspeptin neuron projection to the GnRH neuron cell body in the pubertal period leads to the generation of the GnRH surge, thus resulting in the LH surge and the first ovulation (5).

In 2003, two independent studies reported patients with KISS1R gene inactivation or deletion mutation suffered familial or sporadic forms of idiopathic (or isolated) hypogonadotropic hypogonadism (iHH), which manifested as sexual immaturity and reproductive inability due to the defection of GnRH secretion (9, 106). The phenotype of human *KISS1R* mutation is also present in *kiss1r* knockout mice and rats (9, 107). By contrast, mutations which cause KISS1R hyperactive in humans result in central precocious puberty (CPP). In 2008, a mutation (Arg386Pro) in the kisspeptin receptor gene which results in extended activation of intracellular signaling pathways when responding to kisspeptin was identified in a girl with idiopathic CPP (108). Many studies have confirmed that girls with CPP had higher serum kisspeptin levels than normal controls (109–111). It suggested that the mutant may increase the promoting effects of kisspeptin on GnRH release, thereby speeding up the HPG axis maturation. Another mutation, p.P74S, discovered in a boy with CPP in 2010, has been confirmed to cause kisspeptin more resistant to degradation, suggesting an increased availability of bioactive kisspeptin as another mechanism of precocious puberty (112). In another study, the first compound with the ability to block kisspeptin actions *in vivo* and *in vitro*, namely p234, has been used in pubertal female mammals for experiments. Scientists has demonstrated that the application of p234 in rats for 7 days preceding puberty resulted in delayed puberty onset (113). In contrast, the addition of exogenous kisspeptin led to earlier puberty in rats and monkeys (114, 115). These results imply that kisspeptin plays an integral role in the coordination of pubertal onset.

In summary, kisspeptin is more likely to act as an amplifier of a series of GnRH secretion events rather than the trigger of puberty onset (116). In fact, other partners of kisspeptin neurons such as Substance P, NKA, RFRP-3 and α-MSH also take part in the regulation of puberty timing (70). To be exact, the precise control of puberty onset depends on the interaction and coordination of a large neuronal network.

### 4.2 Kisspeptin and Female Reproduction

Female reproduction is deeply regulated by the hypothalamic–pituitary–ovarian (HPO) axis. The ovary is closely related to follicular maturation, ovulation, corpus luteum formation and secretion of the steroid hormones. The distribution of kisspeptin in the ovary has obvious temporal and spatial specificity, showing its closed relationship with ovarian functions. Being a critical regulator, kisspeptin modulates female reproduction in several aspects, including gonadotropin secretion, follicular development, oocyte maturation and ovulation (17, 44, 117).

#### 4.2.1 The Role in Follicular Development

In human, kisspeptin levels increase from early follicular to preovulatory phase (118). In the process of follicular development, Fernandois et al. showed that kisspeptin affects the primary and secondary follicle recruitment through reducing the FSH receptor (FSHR) expression (119). In both 6- and 10-month-old rats, local administration of kisspeptin into the ovary reduced the number of total antral follicles (including atretic follicles) and the use of kisspeptin receptor antagonist p234 played the opposite role. In an *in vitro* experiment, kisspeptin prevents the increase in FSHR expression produced by ISO (a β-adrenergic agonist), acting as a functional antagonist. Besides, kisspeptin can upregulate the level of serum anti-Müllerian hormone (AMH), which is a vital dimeric glycoprotein in the regulation of follicle development. Produced by preantral and small antral follicles, AMH exerts its regulatory role though attenuating primordial follicle recruitment and changing the sensitivity of follicles to FSH (120, 121). The study found that serum AMH level increased after local administration of kisspeptin and decreased after the use of p234 in 6- and 10-month-old rats. To sum up, kisspeptin may negatively affect the development of preantral follicles by upregulating AMH and downregulating the expression of FSHR in the ovary.

#### 4.2.2 The Role in Oocyte Maturation

It is well known that the preovulatory LH surge triggers the resumption of meiosis and the progression to metaphase II (MII) during each reproductive cycle (122). Besides, the direct effect of kisspeptin on oocyte maturation has been studied in porcine cumulus-oocyte complexes (COCs). Adding kisspeptin to porcine COCs *in vitro* promotes oocyte maturation, suggesting kisspeptin acts on oocytes directly (123). The mechanisms may include upregulating the expression of C-MOS, growth differentiation factor 9 (GDF 9) and bone morphogenetic protein 15 (BMP 15) (124). C-MOS plays a stimulating role in various processes during oocyte maturation, including the meiosis process, normal spindle and chromosome formation, and reactivation of purified maturation promoting factor after first meiosis. Also, GDF 9 and BMP 15 take part in regulating follicle development, oocyte maturation, ovulation, luteinization and other physiological processes (124–127).

It has been found that cumulus granulosa cells (GCs) play a vital role in regulating oocyte maturation. Chakravarthi et al. has observed a remarkable expression of kisspeptin in gonadotropin treated GCs while KISSR in oocytes, suggesting that GC-derived Kisspeptin may have a direct function on oocytes KISS1R to modulate oocyte maturation *via* a MAPK signaling pathway (45, 128, 129). The kisspeptin expressed in GCs is estrogen receptor β (ERβ) dependent since the expression of kisspeptin in GCs is absent in *ERβ* knockout rat ovaries. Consistent with the findings above, the administration of kisspeptin can increase the maturity of oocytes without cumulus cells in both wildtype and *ERβ^null^* rats (45). Therefore, kisspeptin may have a persistent and direct effect on oocytes in an autocrine and paracrine manner.

#### 4.2.3 The Role in Ovulation

Ovulation is a complicated process described as the follicle rupture and oocyte release, which is mediated by the LH surge and is regulated by a series of specific genes (130). At the end of the follicular phase, high levels of estrogen act on AVPV kisspeptin neurons, promoting the release of kisspeptin which then cause the cascade of GnRH surge, LH peak and ovulation (25). The functions of LH peak are achieved by upregulating of COX-2 and producing prostaglandin (131). It has been confirmed that peripheral kisspeptin administration induces ovulation in many species such as rats (61) and ewes (132). In fact, the effect of kisspeptin on ovulation is mainly achieved by increasing the levels of LH and FSH. Subcutaneous administration of kisspeptin markedly elevated plasma FSH and LH levels in 25-day-old female rats (61). In human, the LH pulses increased immediately after an administration of kisspeptin-10 (118). Kisspeptin-54 induced ovulation in mice by stimulating precisely timed endogenous LH release of consistent amplitude and duration (133). Both the expression of ovarian Kiss1 mRNA and the ovulation efficiency in rats could be reduced by the administration of COX-2 inhibitor or COX non-selective inhibitor, indicating that the upregulation of COX-2 may act on the expression levels of kisspeptin to induce LH peak (19).

The role of ovarian kisspeptin in ovulation may not be indispensable because in *Kiss1r* knockout mice, standard gonadotropin priming could induce ovulation (9), indicating that the ovarian kisspeptin signaling is not nexessary for ovulation. However, although the oocyte quality between neuron-specific *Kiss1* and *Kiss1R* knockout mice and wild type mice shows little difference, the knockout mice presented obviously fewer ovulated oocytes and corpora lutea. This suggests the GnRH plus gonadotropin stimulation is not sufficient to reverse the loss of function due to *Kiss1r* knockout (134).

#### 4.2.4 The Role in Reproductive Behaviors

Lordosis is an important posture adopt by non-primate female mammals in response to male mounting, characterized by ventral arching of the spine and elevation of the hips to facilitate penile penetration (135). A study has shown that female *Kiss1*-knockout mice were unable to exhibit normal lordosis behavior despite estrogen and progesterone replacement (136), while kisspeptin 10 injection in *Kiss* ^−/−^ females robustly stimulated lordosis behavior. Optogenetic activation of RP3V kisspeptin neurons caused strong firing and provoked the sexual behavior of lordosis, showing that RP3V kisspeptin neurons are essential for lordosis (136). The effect of kisspeptin is closely associated with NO, which is potentially a key transmitter downstream of kisspeptin neurons. Furthermore, this study also found that kisspeptin neurons induce mate preferences in female mice driven by olfaction *via* GnRH neurons. Together, these observations illustrate the crucial role of kisspeptin in controlling the sexual behaviors in female mice. However, ovariectomized *Kiss1r*-knockout mice showed normal female sexual behaviors such as lordosis after hormone replaced (with estrogen and progesterone) treatment (137). Consistent with this result, lordosis behavior is also unaffected in an additional transgenic mouse model which completely lacks GnRH immunoactivity in adulthood. These surprising results implied that the kisspeptin receptor is not necessary for normal lordosis behavior in female mice while sufficient sex steroid replacement is. In other words, kisspeptin-mediated lordosis is not dependent on GnRH signaling (136). One reasonable hypothesis is that kisspeptin-mediated-lordosis is facilitated *via* NPFF receptors as both NPFFR1 and NPFFR2 have been found in brain areas which are important in female sexual behavior (136, 138). However, the hypothesis has not been confirmed by any research at present.

### 4.3 Kisspeptin and Male Reproduction

Spermatogenesis is a complex process in testis in which the spermatogonia develop into male gametes through the adjustment of mitotic, meiotic and differentiation events (20). Each component of testis and the overall function are controlled by hypothalamic-pituitary-testicular axis. As the gatekeeper in HPG axis, GnRH induces the production of sex steroid by Leydig cells and the processes of spermatogenesis through LH and FSH released by the pituitary. Many studies have demonstrated the distribution of kisspeptin and its receptor proteins in the testes in multiple species including non-mammalian vertebrates such as frogs, fish and mammals such as rodents, goats and monkeys (21, 139–141). The major producer of kisspeptin is considered to be Leydig cell since scientists have detected the substantial expression of *Kiss1* mRNA or protein in Leydig cells in various mammalian and non-mammalian species such as mice, horses, goats and frogs (141–144). Besides, kisspeptins were also expressed in epididymis. Mele et al. observed the differential kisspeptin expression in caput and cauda rat epididymis, while it is still not clear whether epidermal kisspeptins assist with maturation of sperm (140). In mammals, gene expression profiling showed that the initiation of kisspeptin/kiss1r expression in mouse testis and the formation of spermatozoa happened at the same time, indicating there is a connection between spermatogenesis and testicular kisspeptin/kiss1r system (145). In general, testicular kisspeptin system may act in the regulation of Leydig cell activity, spermatogenesis, and sperm function.

As for steroidogenesis, it has been reported that testosterone levels decreased after the administration of different doses of kp-10 (25). Other studies reported that kp-10 dramatically increased total testosterone plasma levels in the acute phase while chronic administration was associated with a decrease. In adult Wistar rats, chronic subcutaneous administration of kp-54 (50 nmol/d) in long term (13 days)decreased testicular weight, induced seminiferous tubules to degenerate (146). Continuous subcutaneous injection of kp-54 led to testicular degeneration after only 12 hours, at the time when LH and FSH were still considerably increased (147). Based on the results, the reduction of testosterone was attributed to central hyper-stimulation of the HPG axis, which needs to be further confirmed. However, some studies got different results. For example, a study reported that neither acute nor chronic injection of 50 nmol/kg kisspeptin-10 failed to induce degeneration of rat seminal tubules (148). According to the difference in results above, it is difficult to draw some exact conclusions at present.

In the process of spermatogenesis, *in vivo* kisspeptin administration accelerates spermatogenesis until sperm production in fish (149). During the late stage of spermatogenesis, the administration of kp-13 regulated sperm motility and caused momentary sperm hyperactivation, with a slow raise of sperm intracellular Ca^2+^concentration in human, suggesting the effects of kisspeptin to the sperm is achieved *via* the increase of Ca^2+^ levels (150). The use of p234 blocked the above effects of kp-13, indicating that the role of kisspeptin in human spermatogenesis is direct. Similar results were also obtained in rodents (22).

In terms of reproductive behaviors, male mice with *Kiss1r* knocked out are unable to show normal male sexual behaviors, such as mount, thrust and ejaculation (137). However, the failure can be compensated by testosterone, although the proportion of male ejaculation in testosterone substituted *kiss1r* knockout mice was lower than that in the wild type (137). Recent studies have concentrated on the positive role of the amygdala (MeA) kisspeptin system in modulating sexual behaviors (151, 152). It is reported that *KISS1* mRNA expression in male MePD (posterodorsal medial amygdala) is higher than that in female (153). After exposing to female urine, MeA kisspeptin neuronal activity in male mice showed a doubled increase with an accompanied LH surge, indicating the important role of the amygdala kisspeptin system in olfactory-reproductive pathways (154). Moreover, the results triggered by intra-MePD and intra-cerebral injection of kp-10 are not exactly the same, the intra-MePD injection can induce penile erection in addition to inducing LH release, revealing that the enhancement of erectile function is achieved through the direct action of kisspeptin on MePD (152). However, in a recent study, DREADD (designer receptor exclusively activated by designer drugs)-mediated activation of MePD kisspeptin neurons in male mice caused notable levels of sexual behaviors such as mounts, intromissions, and ejaculations in the presence and absence of DREADDs-induced activation of these kisspeptin neurons, suggesting the role of MePD kisspeptin neurons is limited (151, 155). Also, in humans, male patients with *KISS1R* mutations successfully became fertile after treated with exogenous hormonal (156). These interesting findings showed that kisspeptin receptors may be important but not essential for male mammalian spermatogenesis and certain reproductive behaviors (157). Since there is not much research on it, it awaits further delineation.

## 5 Kisspeptin and Reproductive Diseases

As kisspeptin is an upstream regulator of GnRH neurons, it is related to some diseases associated with impaired HPG axis. It is widely known that the mutation of *KISS1R* gene lead to abnormal puberty onset, manifesting with CPP or iHH. A survey in Korea has shown that girls with CPP had higher serum kisspeptin levels compared with same aged prepubertal controls (111). The relevant contents have been mentioned in part 3.1.

PCOS is a common female reproductive endocrine disease whose typical clinical manifestations include infertility, ovulatory dysfunction, hyperandrogenism and so on (44). PCOS women show elevated LH/FSH, which indicates the impaired feedback of GnRH pulses (158). Due to the role of kisspeptin in LH release, the kisspeptin level in PCOS patients is speculated to be higher. This hypothesis has been supported by a large number of studies in human, most of which showed that the serum kisspeptin level of patients with PCOS is significantly higher than that of healthy controls (159, 160). Also, the role of ovarian kisspeptin has been confirmed in a previous study. The administration of kisspeptin in woman who accepted *in vitro* fertilization was found to change gene expression in granulosa lutein cells remarkably, triggering a higher expression of FSH and LH receptor, and higher expression of genes associated with ovarian steroid synthesis and action (161). In animal models, the expression kisspeptin only increased in PCOS models with increased LH and normal weight, while in androgen induced models kisspeptin expression were significantly suppressed (158, 162). In general, kisspeptin affects LH secretion and steroidogenesis through central and periphery pathways in PCOS women, while it needs more surveys and studies in both human and animals to illustrate and confirm its detailed role.

Hyperprolactinemia is another reproductive endocrine disease characterized by high level of serum prolactin (PRL) and hypogonadotropic anovulation which leads to infertility (163). It has been reported that kisspeptin neurons are the targets of PRL, as PRL receptors are expressed in most kisspeptin neurons but few GnRH neurons (164). Ovariectomized rats which accepted exogenous PRL were observed to have higher expression of phosphorylated signal transducer and activator of transcription 5 (pSTAT5) and reduced kisspeptin expression in ARC kisspeptin neurons, suggesting the direct effect of PRL on ARC kisspeptin neurons (163, 165). It seems that PRL acts on ARC kisspeptin neurons and results in reduction of GnRH levels, thereby leads to the decrease of LH levels and ovulation inhibition.

Currently kisspeptin is related to various reproductive diseases although the detailed role remains to be explored. Understanding the role of kisspeptin in the occurrence of these reproductive endocrine diseases will provide a theoretical basis for the treatment of these diseases in the future.

## 6 Potential Roles of Kisspeptin in Clinical Application

In recent years, many attempts have been made to explore the possibility of kisspeptin as a new diagnostic marker or therapeutic option. In humans, the plasma level of kisspeptin (kisspeptin-54) increases dramatically throughout pregnancy, making it possible to detect early pregnancy by measuring plasma kisspeptin concentrations (166). Also, because kisspeptin is produced by trophoblasts and trophoblast invasion is underway 5 days after blastocyst transplantation, plasma kisspeptin concentrations during the peri-implantation period may reflect the early developmental events associated with pregnancy outcome (167). In a comparative study, Sullivan-Pyke et al. measured serum kisspeptin in 20 women with 6-10 weeks of intrauterine pregnancy (IUP) and 20 women who suffered spontaneous abortion (SAB) at a similar time. They found the median serum kisspeptin levels were significantly higher in IUP women (1.50ng/ml) than in SAB women (0.20ng/ml), indicating that kisspeptin is detectable in serum in early pregnancy and can discriminate SAB from IUP (168). Also, as mentioned above, girls with CPP had higher serum kisspeptin levels compared with healthy girls (109–111). However, serum kisspeptin levels are not able to become a single diagnostic tool because the evident overlap limits its use, while it may still be useful as an adjunctive tool in the diagnosis of CPP.

As for therapeutic option, kisspeptin is found to have potential in stimulating oocyte maturation and inducing ovulation in infertile women. In 2014, a clinical study found that single administration of kisspeptin-54 induced female egg maturation in women who accepted *in vitro* fertilization (169), suggesting its potential application in treating women with infertility. Kisspeptin and its agonist are also be regard as potential therapeutic options of some reproductive diseases. In a clinical study, repeated administration of kp-54 successfully induced ovulation in two out of seven women with PCOS (170). Abbara et al. compared the therapeutic effect of nanopeptide KISS1R agonist MT-602 and kisspeptin 54 in PCOS women and found both MVT-602 and kp54 induced a LH peak with similar amplitude (171). Recent studies also reported the application of kisspeptin as a future therapeutic option in treatment of hyperprolactinemia. The administration of kisspeptin successfully caused LH pulses *via* stimulating GnRH in women with hyperprolactinemia (172). In another study, the use of kisspeptin induced recover of gonadotropin secretion and ovarian cyclicity (173). In addition, based on the fact that high dose of kisspeptin leads to desensitization of HPG axis, kisspeptin may be applied in the treatment of sex hormone-dependent malignancies. For example, prostate cancer is a kind of androgen dependent malignancy and the current primary treatment is androgen deprivation therapy (ADT) (174). Two animal studies have confirmed that chronic administration of the kisspeptin analog, TAK-448 caused stronger inhibiting effect of HPG axis than GnRH analog and obviously suppressed testosterone and LH release, indicating its great anti-tumor growth potential (175, 176).

## 7 Conclusion and Future Directions

**Figure 3:**
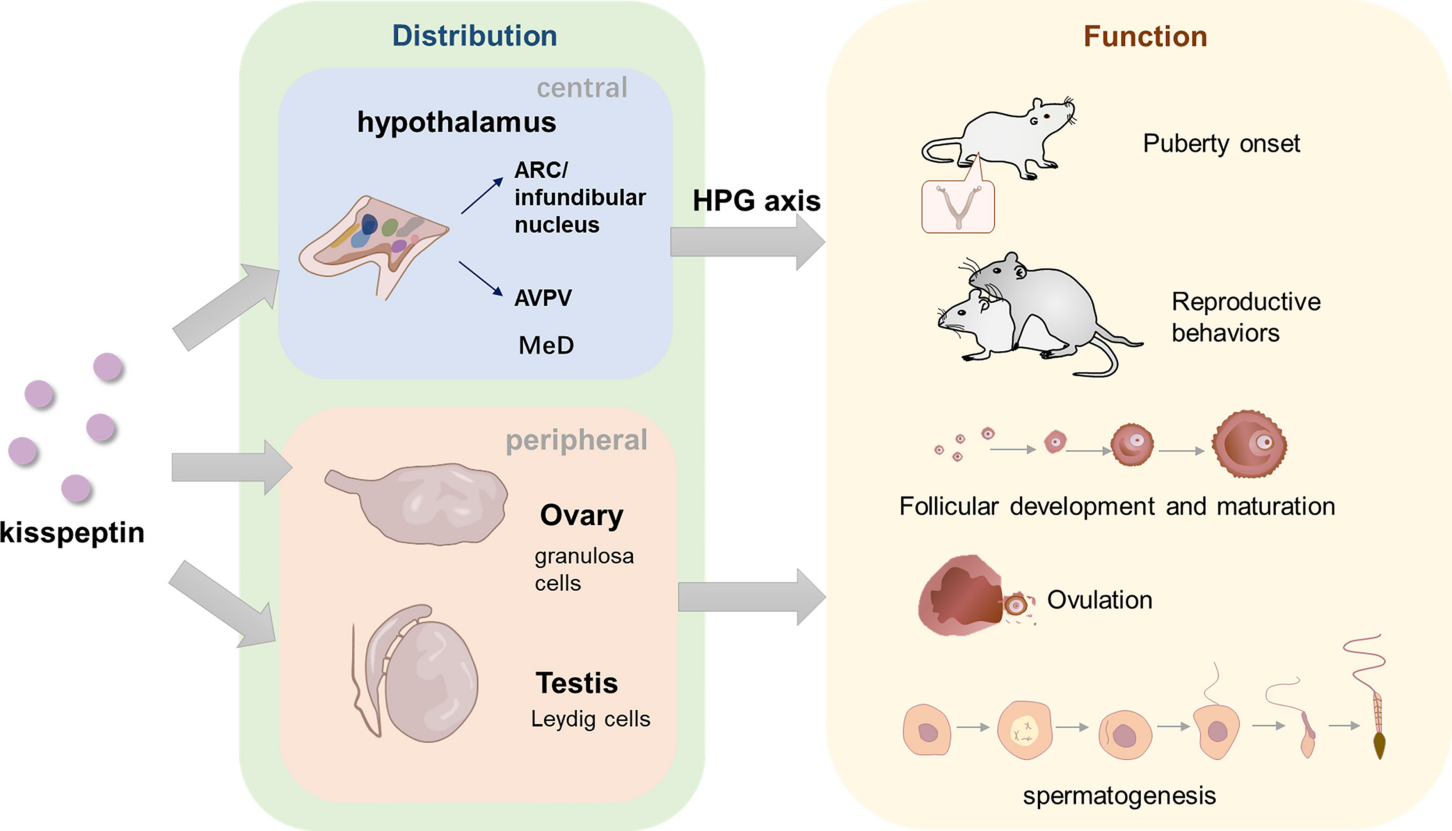
The distribution of kisspeptin in central and peripheral areas and its role in reproduction. In hypothalamus, kisspeptins are located in two main neuron populations, ARC/infundibular nucleus and AVPV. In the peripheral areas, kisspeptin are mainly discovered in ovary and testis. GCs are the main cell which product Ovarian-derived kisspeptin, while Leydig cells are considered to product testicular kisspeptin. Kisspeptins are also found in other organs such as pancreas, liver, placenta, etc. (not depicted in the figure). Kisspeptin in the hypothalamus functions through HPG axis, while gonads-derived kisspeptin might act directly in a autocrine-paracrine manner. In terms of the function, kisspeptin is likely to act as an amplifier of a series of GnRH secretion events, which are vital for normal puberty onset. Central and peripheral kisspeptins exert a role in various processes of female and male reproduction, maintaining the normal progress of follicular development, oocyte maturation, ovulation in female and spermatogenesis in male. In addition, kisspeptin is also essential in both male and female reproductive behaviors, regulating mount, ejaculation and thrust in male and lordosis in female.

The discovery of kisspeptin was a milestone in the field of reproductive biology. The last two decades have seen an explosion in kisspeptin literature, which elucidated of the pivotal role of kisspeptin in the control of HPG axis. Kisspeptin acts upstream of GnRH and thus regulates the secretion of LH and FSH, following paracrine promoting and inhibiting inputs from NKB and Dyn, signals directly to GnRH neurons to control pulsatile GnRH secretion, thus regulates the secretion of LH and FSH. Through the G-protein-coupled receptor, KISS1R, kisspeptin acts in the onset of puberty and maintenance of mammalian fertility. Although there is some difference between sexes in neuroanatomy and function, the functions of kisspeptin are apparent in both male and female. On the one hand, kisspeptin in hypothalamus acts on HPG axis, regulating reproduction processes through secretion of LH and FSH. On the other hand, periphery kisspeptin has a direct effect on the gonads in an autocrine-paracrine manner. In female, ovarian kisspeptin negatively regulate preantral follicular development and increase the maturity of oocyte. In male, kisspeptin are closely associated with spermatogenesis although the detailed role is not completely clear. Kisspeptin also regulates reproductive behaviors in both male and female including lordosis in female and mount, thrust or ejaculate in male **(** **Figure 3** **)**.

The large number of experiments on mammals in recent years has significantly improved our understanding of the role of kisspeptins and related mechanisms. Modern approaches such as optogenetics, mathematical models and *in vivo* calcium imaging have made huge contribution to the solution of long-existing questions such as the mechanism of negative and positive feedback and the functions of kisspeptin/KISS1R system in female reproduction. However, there are still some questions which need further exploration. For instance, although many new advances have been made in male reproduction, the role of kisspeptin in steroidogenesis, sperm function and reproductive behaviors are not completely clear. The different results in experiments need further researches to explain. Recently the differential expression of kisspeptin receptor in epidermis was discovered, while its specific effect on sperm maturation is not confirmed yet. Besides, the potential effects of kisspeptin on testicular germ cells and somatic cells were studied by tissue and cell culture experiments, and its role *in vivo* is not fully understood (157). Also, most current studies were only conducted on animal models, thus more powerful evidence are needed to prove the relevance of these findings to human. In addition to the two main populations of kisspeptin neurons, a small number of kisspeptin neurons was also found in the limbic system of the brain (such as amygdala, the lateral septum, the dorsomedial and ventromedial hypothalamic nuclei, etc.). The function and precise lineage of these kisspeptin populations are also worthy of further exploration (70, 102). The development of new technologies will help bring new ideas about the unknown field.

Based on the essential effects of kisspeptin in reproductive axis summarized in this review, kisspeptin and its analogues may be used for diagnostic and therapeutic goals. This needs more data, more translational work and larger studies to expand our knowledge of kisspeptin. In conclusion, a complete understanding of the expression, function, and potential molecular mechanisms of kisspeptin/KISS1R in the HPG axis and its role in reproduction will bring new inspiration to the diagnosis, treatment and prevention of some reproductive diseases.

## Author Contributions

QX, YK wrote the article and performed all of the necessary literature searches and data compilation. YX, CW, JL, CZ, CY performed the necessary literature searches and data compilation. HZ revised the article and give valuable suggestions. DH designed the review, reviewed it, and approved the submitted manuscript. All authors have read and approved the final manuscript.

## Funding

This work was supported by the National Natural Science Foundation of China (NO. 81771575) and National Key Research & Developmental Program of China (2018YFC1003900).

## Conflict of Interest

The authors declare that the research was conducted in the absence of any commercial or financial relationships that could be construed as a potential conflict of interest.

## Publisher’s Note

All claims expressed in this article are solely those of the authors and do not necessarily represent those of their affiliated organizations, or those of the publisher, the editors and the reviewers. Any product that may be evaluated in this article, or claim that may be made by its manufacturer, is not guaranteed or endorsed by the publisher.
